# *QuickStats:* Percentage* of Adults Who Are Very Worried About Ability to Pay Medical Bills if They Get Sick or Have an Accident,**^†^** by Home Ownership**^§^** and Age Group — National Health Interview Survey, United States, 2019**^¶^**

**DOI:** 10.15585/mmwr.mm7022a4

**Published:** 2021-06-04

**Figure:**
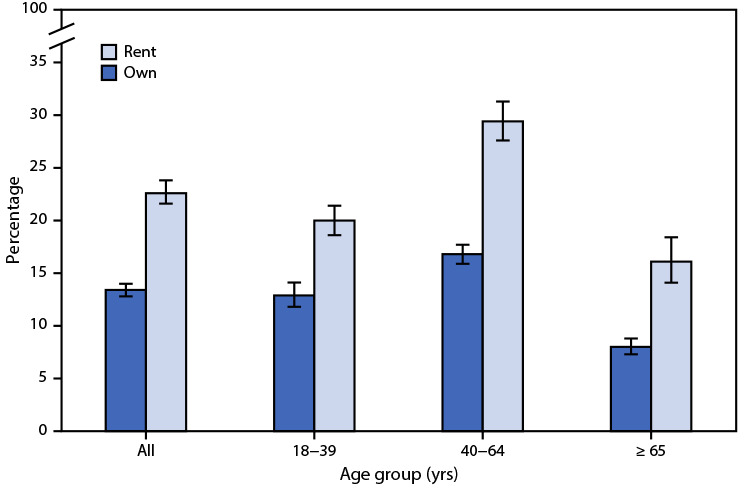
In 2019, 22.6% of renters were very worried about their ability to pay their medical bills if they get sick or have an accident, compared with 13.4% of homeowners. For each age group, renters were more likely than homeowners to be very worried about paying their medical bills: 20.0% compared with 12.9% among those aged 18–39 years, 29.4% compared with 16.8% among those aged 40-64 years, and 16.1% compared with 8.0% among those aged ≥65 years.

**Source:** National Health Interview Survey, 2019. https://www.cdc.gov/nchs/nhis.htm

